# Spatial and Vertical Variations and Heavy Metal Enrichments in Irrigated Soils of the Syr Darya River Watershed, Aral Sea Basin, Kazakhstan

**DOI:** 10.3390/ijerph16224398

**Published:** 2019-11-11

**Authors:** Long Ma, Jilili Abuduwaili, Zhassulan Smanov, Yongxiao Ge, Kanat Samarkhanov, Galymzhan Saparov, Gulnura Issanova

**Affiliations:** 1 State Key Laboratory of Desert and Oasis Ecology, Xinjiang Institute of Ecology and Geography, Chinese Academy of Sciences, Urumqi 830011, China; malong@ms.xjb.ac.cn (L.M.); zhassulan.smanov1307@gmail.com (Z.S.); geyx@ms.xjb.ac.cn (Y.G.); skgeo@mail.ru (K.S.); 2 Research Center for Ecology and Environment of Central Asia, Chinese Academy of Sciences, Urumqi 830011, China; 3 University of Chinese Academy of Sciences, Beijing 10049, China; 4 Kazakh Research Institute of Soil Science and Agrochemistry Named after U. U. Uspanov, Almaty 050060, Kazakhstan; saparov.g@mail.ru; 5 Faculty of Geography and Environmental Sciences, Al–Farabi Kazakh National University, Almaty 050040, Kazakhstan; gul_nur.777@mail.ru

**Keywords:** heavy metals, enrichment factor, human health risk, irrigated soils, Syr Darya River, Kazakhstan

## Abstract

In the Syr Darya River watershed, 225 samples from three different layers in 75 soil profiles were collected from irrigated areas in three different spatial regions (I: *n* = 29; II: *n* = 17; III: *n* = 29), and the spatial and vertical variation characteristics of potentially toxic elements (Cd, Co, Cu, Ni, and Zn) and a metallic element (Mn) were studied. The human health risks and enrichment factors were also evaluated in the Syr Darya River watershed of the Aral Sea Basin in Kazakhstan. There were significant differences in the contents of heavy metals in the different soil layers in the different sampling regions. Based on element variation similarity revealed by hierarchical cluster analysis, the elemental groupings were consistent in the different layers only in region I. For regions II and III, the clustered elemental groups were the same between surface layer A and B, but differed from those in the deep layer C. In sampling region I, the heavy metals in surface soils were significantly correlated with the ones in deep layers, reflecting that they were mainly affected by the elemental composition of parent materials. In region II, the significant correlations only existed for Cu, Mn, and Zn between the surface and deep layers. The similar phenomenon with significant correlation was also observed for heavy metals in sampling region III, except for Cd. Finally, enrichment factor was used to study the mobilization and enrichment of potentially toxic elements. The enrichment factors of Zn, Cu, and Cd in surface layer A that were greater than 1.5 accounted for 1.16%, 6.79%, and 24.36% of sampling region I, respectively. In sampling region II, the enrichment factors of Zn, Cu, Cd, and Co that were greater than 1.5 accounted for 0.03%, 4.76%, 0.54%, and 9.03% of the total area, respectively. In sampling region III, only the enrichment factors of Zn, Cu, and Cd that exceeded 1.5 accounted for 0.24%, 4.90%, and 6.89% of the total area, respectively. Although the contents of the heavy metals were not harmful to human health, the effects of human activities on the heavy metals in the irrigated soils revealed by enrichment factors have been shown in this study area.

## 1. Introduction

Human environmental modifications have greatly increased over the past century, and human land use has altered the structure and functioning of ecosystems [1,2,3]. Soil quality is critical for human health, and it is not surprising that soil research has increased exponentially in recent decades [4,5]. The geochemical composition of soils is a direct indicator of soil quality and reflects not only the soil’s natural condition but also the influences of human activities [6,7,8]. Soil heavy metals have many sources, including the parent material, atmospheric deposition, and various human activities such as generate suspended dust and sewage drainage, fertilizers, and irrigation water [9,10]. Soil pH [11,12], organic matter [13,14], soil texture [15] are the main factors controlling the distribution of heavy metals. A great deal of research has focused on land use [16,17] and soil salinization in central Asia [18,19], but there are no studies on the geochemical composition and levels of heavy metals in the soils in this area.

**Figure 1:**
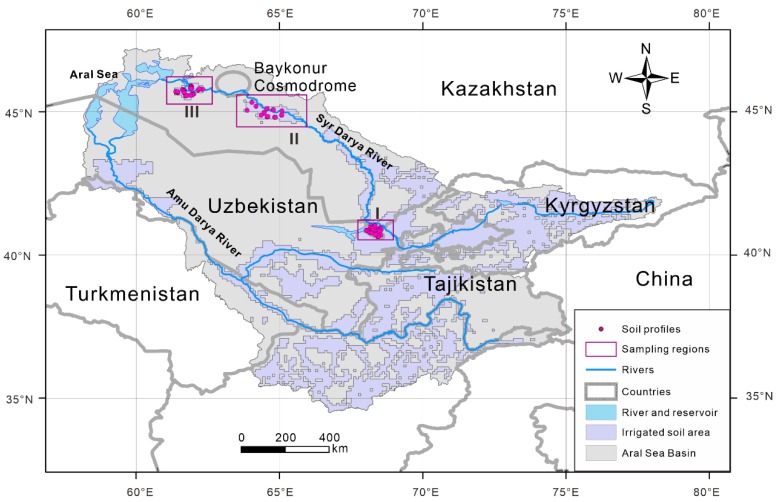
Locations of the study regions (I, II, and III) and distributions of the soil sampling profiles (I: *n* = 29; II: *n* = 17; III: *n* = 29). The boundaries of the irrigation areas are from the literature [31]. The geographical coordinates of the soil profiles are shown in Table S1 in the supplementary file.

The Syr Darya River is the second largest river in the Aral Sea Basin; most of the research on this system has focused on the water source of the Syr Darya River [20,21], changes in the water volume of the Syr Darya River and its influencing factors [22,23,24,25], water resource countermeasures [26,27], and climate change effects [28,29,30]. The irrigated land in Kazakhstan is mainly distributed along the Syr Darya River [31] (Figure 1). On the one hand, the heavy metals in soils are directly related to public health and indirectly affect societal and economic development. However, previous studies have paid relatively less attention to variabilities in the heavy metals in the farmland soils in this region. On the other hand, disasters in the Aral Sea in central Asia have had a profound impact on the environment. The chemical substances in salt storms from the Aral Sea [32,33] are indirectly derived from the erosion of surface materials, and the heavy metals in salt storms have important impacts on the ecological environment [34]. The lack of research on the characteristics and distribution of heavy metals in farmland soils has a far-reaching influence on the sustainable development of agriculture in central Asia under the background of global change; indirectly, the study of the material sources of salt dust storms in the Aral Sea is affected, which is an important research gap regarding comprehensive environmental regulations of the Aral Sea.

The aims of this study were to answer the following scientific questions. What are the vertical and spatial distribution characteristics of the heavy metals in irrigated soils in the region of the Syr Darya River? Is there an inheritance relationship between the heavy metals in different soil layers in the soil profiles? Do farming activities by humans significantly change the content of heavy metals in the farmland soils?

## 2. Materials and Methods 

### 2.1. Sampling and Laboratory Analysis

For the sampling region I, the bedrocks are mainly composed of mixed sedimentary rocks and a small number of unconsolidated sediments [35], and the soils in the study area are composed of Anthrosols and a small number of Calcisols from Harmonized World Soil Database (v 1.2) [36]. In this region, annual total precipitation was 340.1 mm (multi-year annual average from 1901–2018), and mean annual temperature of 15.1 °C (multi-year monthly average from 1901–2018) [37]. For the sampling region II, the bedrocks are mainly composed of unconsolidated sediments and a small number of mixed sedimentary rocks [35], and the soils in the study area are composed of Gleysols and a small number of Solonchaks [36]. It is the typical temperate continental arid climate, with annual total precipitation of 128.7 mm (multi-year annual average from 1901–2018), and mean annual temperature of 9.8 °C (multi-year monthly average from 1901–2018) [37]. The bedrocks in the sampling region III are same as the region II [35], and the soils in the study area are Calcisols [36]. It is the typical temperate continental arid climate, with annual total precipitation of 119.5 mm (multi-year annual average from 1901–2018), and mean annual temperature of 8.9 °C (multi-year monthly average from 1901–2018) [37].

From three relatively independent irrigation regions (I, II, and III, Figure 1) along the Syr Darya River, 75 soil profiles were excavated to a depth of 100 cm (I: *n* = 29; II: *n* = 17; III: *n* = 29), and three soil samples were collected from three different layers in each profile (0–20, 21–50, and 51–100 cm). In total, 225 soil samples were air-dried and sieved through a 2 mm sieve. Soil pH (pH electrode meter, PHS–3C, LeiCi Co. Ltd., Shanghai, China) was measured of deionized water suspension of 1:2.5 (*w/v*), electrical conductivity (EC; conductivity meter, DDS–307, LeiCi Co. Ltd., Shanghai, China) was determined in deionized water suspension of 1:5 (*w/v*) [38,39]. The contents of potentially toxic elements (Cd, Co, Cr, Cu, Ni, and Zn) and a metallic element (Mn) were analyzed with the strong acid (HNO_3_–HClO_4_) pseudototal digestion method [40] and determined by inductively coupled plasma mass spectrometry (ICP–MS). The abovementioned analyses were performed at the Research Center for Ecology and Environment of Central Asia (Almaty).

### 2.2. Statistical Analysis

Hierarchical cluster analysis (HCA) is the most commonly used environmental analysis method and reveals variable groups based on their similarities [41,42,43,44,45]. In this study, HCA was used to evaluate the differences and similarities of the heavy metals in different soil layers, and the grouping results were presented in a dendrogram [46]. Linear regression was used to study the relationship of the heavy metals in the different soil layers [47,48].

### 2.3. Enrichment Assessments

The enrichment factor (EF) metric has been used to assess the enrichment extent of potentially toxic elements (Cd, Co, Cr, Cu, Ni, and Zn) in soils and can also be used to distinguish between anthropogenic sources and natural sources [44,49,50]. EF was calculated by dividing the ratio of the content of a potentially toxic element to its background content by the ratio of the content of a reference element to its background content [51]:

(1) $$\mathrm{EF}=\frac{{\left(C_{i}/C_{ref}\right)}_{sample}}{{\left(B_{i}/B_{ref}\right)}_{background}}$$

where *C_i_* is the content of a potentially toxic element in the soil (mg/kg); *C_ref_* is the background content of the potentially toxic element (mg/kg); *B_i_* is the content of a reference element (mg/kg) within the same layer as *C_i_*; and *B_ref_* is the background content of the reference element (mg/kg). In this study, a metallic element (Mn) was used as the reference element because the concentration of Mn in the earth’s crust is relatively high and more stable [52,53,54,55].

### 2.4. Health Risk Assessment

Human health risk assessments are widely used to evaluate the health risks of heavy metals in soils [55,56,57]. A noncarcinogenic hazard index was calculated for three exposure pathways (i): Ingestion, direct ingestion, and indirect ingestion via produce consumption; dermal contact; and inhalation, inhalation of resuspended soil particulates by nose or mouth [58]. For exposure pathway (i), noncarcinogenic hazards, such as the hazard quotient (HQi), were calculated with the rate of the corresponding reference dose for the exposure pathway (RfDi):

(2) $$HQ_{i}=ADD_{i}/RfD_{i}$$

(3) $$ADD_{ing}(mg\cdot kg^{-1}\cdot day^{-1})=C_{x}\times \frac{IngR\times ExF\times ED}{BW\times AT}\times 10^{-6}$$

(4) $$ADD_{inh}(mg\cdot kg^{-1}\cdot day^{-1})=C_{x}\times \frac{InhR\times ExF\times ED}{PEF\times BW\times AT}$$

(5) $$ADD_{derm}(mg\cdot kg^{-1}\cdot day^{-1})=C_{x}\times \frac{SA\times SL\times ABS\times ExF\times ED}{BW\times AT}\times 10^{-6}$$

where *ADD_ing_* is the average daily intake via ingestion; *ADD_inh_* is the average daily intake via inhalation; *ADD_derm_* is the average daily intake via dermal absorption; *C_x_* is the potentially toxic element content (mg kg^−1^). In this study, the maximum values of potentially toxic elements in the surface layers were chosen as *C_x_*; *IngR* is the ingestion rate (200 mg day^−1^) [59]; *ExF* is the exposure frequency (350 day year^−1^) [60]; ED is the exposure duration (30 a) [60]; *BW* is body weight (70 kg) [60]; *AT* is the exposure time (*AT* = 365 × *ED*); *InhR* is the inhalation rate (12.8 m^3^ day^−1^) [61]; *PEF* is particle emission factor (1.36 × 10^9^ m^3^ kg^−1^) [59]; *SA* is the exposed skin area (4350 cm^2^) [61]; *SL* is the skin adherence factor (0.2 mg cm^−2^ day^−1^); and *ABS* is a dimensionless dermal absorption factor (*ABS* = 0.001; for As, *ABS* = 0.03 [59]). 

The hazard index (HI) was calculated as follows:

(6) $$HI=\sum_{i=1}^{3}HQ_{i}$$

If HI < 1 or HQ < 1, there are no noncarcinogenic risks; otherwise, the state of HI > 1 or HQ > 1 inferred that noncarcinogenic effects exist [62].

## 3. Results and Discussion

### 3.1. Statistical Characteristics of the Soil Geochemical Indicators

**Figure 2:**
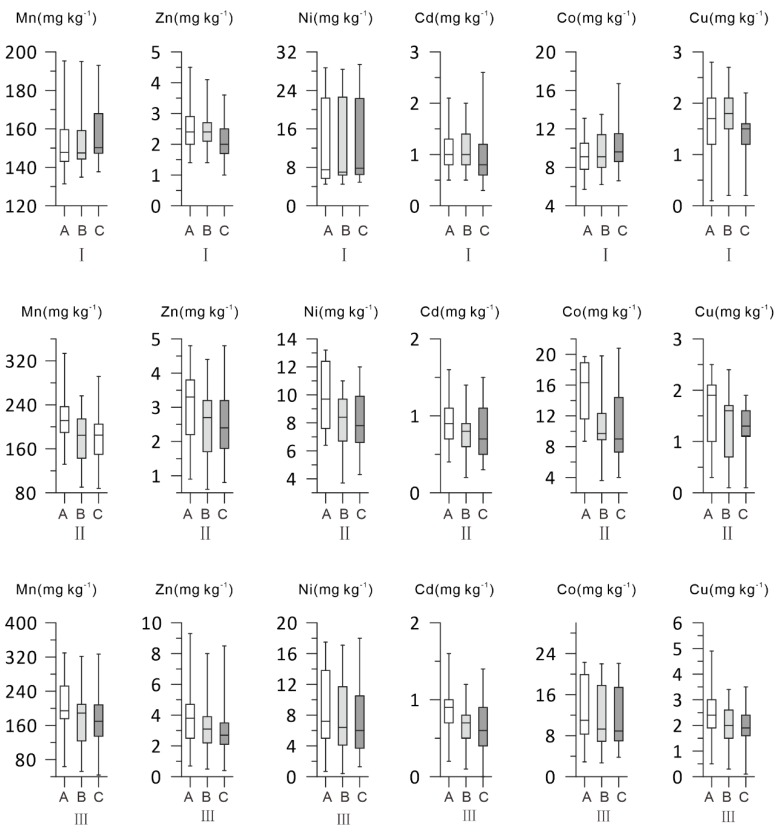
The vertical box plots display the minimum, maximum, median, lower quartile, and upper quartile data of the heavy metals in the three sampling regions (I: *n* = 29; II: *n* = 17; III: *n* = 29). A: The 0–20 cm soil layer; B: The 21–50 cm soil layer; and C: The 51–100 cm soil layer.

The pH, EC, and contents of heavy metals of the soils differed in the three sampling regions. There were also great differences in the pH, EC, and contents of heavy metals in the different soil layers in a single sampling region (Figure 2 and Table S2 in the supplementary file).

In sampling region I, the surface soil (0–20 cm) pH ranged from 7.5 to 8.4, with an average of 8.0. The EC ranged from 62.2–1222 μS cm^−1^, with an average of 414.2 μS cm^−1^. The average contents of Zn, Cu, Cd, Ni, Co, and Mn were 2.5, 1.7, 1.1, 13.6, 9.4, and 153.4 mg kg^−1^, respectively. The pH values of the soils in the middle layer (21–50 cm) ranged from 7.2−8.5, with an average of 8.0. The EC ranged from 104.4–1369 μS cm^−1^, with an average of 476.9 μS cm^−1^. The average contents of Zn, Cu, Cd, Ni, Co, and Mn were 2.5, 1.7, 1.1, 14.1, 9.6, and 154.2 mg kg^−1^, respectively. The pH values of the soils in the deepest layer (51–100 cm) ranged from 7.4 to 8.8, with an average of 8.0. The EC ranged from 61.4–1417 μS cm^−1^, with an average of 451.3 μS cm^−1^. The average contents of Zn, Cu, Cd, Ni, Co, and Mn were 2.1, 1.4, 0.9, 14.1, 10.0, and 157.1 mg kg^−1^, respectively. 

For sampling region II, the surface soil (0–20 cm) pH ranged from 7.6 to 8.5, with an average of 8.0. The EC ranged from 215–2660 μS cm^−1^, with an average of 1231.4 μS cm^−1^. The average contents of Zn, Cu, Cd, Ni, Co, and Mn were 3.1, 1.6, 0.9, 9.9, 15.0, and 216.4 mg kg^−1^, respectively. The pH values of the soils in the middle layer (21–50 cm) ranged from 7.7–8.5, with an average of 8.1. The EC ranged from 179.5–2330 μS cm^−1^, with an average of 845.4 μS cm^−1^. The average contents of Zn, Cu, Cd, Ni, Co, and Mn were 2.5, 1.2, 0.8, 8.2, 10.6, and 174.3 mg kg^−1^, respectively. The pH values of the soils in the deepest layer (51–100 cm) ranged from 7.8 to 8.6, with an average of 8.1. The EC ranged from 148.8−1387 μS cm^−1^, with an average of 570.8 μS cm^−1^. The average contents of Zn, Cu, Cd, Ni, Co, and Mn were 2.5, 1.2, 0.8, 8.2, 11.1, and 179.0 mg kg^−1^, respectively.

For sampling region III, the surface soil (0–20 cm) pH ranged from 7.3 to 8.9, with an average of 8.1. The EC ranged from 149.4–5020 μS cm^−1^, with an average of 1547.5 μS cm^−1^. The average contents of Zn, Cu, Cd, Ni, Co, and Mn were 4.0, 2.4, 0.8, 8.8, 13.3, and 199.2 mg kg^−1^, respectively. The pH values of the soils in the middle layer (21–50 cm) ranged from 7.2–8.7, with an average of 8.2. The EC ranged from 165–3540 μS cm^−1^, with an average of 1007 μS cm^−1^. The average contents of Zn, Cu, Cd, Ni, Co, and Mn were 3.4, 2.0, 0.6, 7.7, 11.8, and 173.0 mg kg^−1^, respectively. The pH values of the soils in the deepest layer (51–100 cm) ranged from 7.9 to 8.8, with an average of 8.3. The EC ranged from 134–3270 μS cm^−1^, with an average of 937.2 μS cm^−1^. The average contents of Zn, Cu, Cd, Ni, Co, and Mn were 3.2, 2.0, 0.6, 7.5, 11.8, and 170.9 mg kg^−1^, respectively.

As can also be seen from Figure 2 and Table S2 in supplementary file, the content of Cu, Cd, and Co in sampling region III is higher than that in sampling regions I and II. According to the published studies [63], the content of Cu, Cd, and Co in water samples from the lower reaches of the Syr Darya River is also higher than that in the upper reaches. This may reflect that some parts of the heavy metals in the soils of the sampling region III may be derived from the transport of heavy metals from the upper reaches of the Syr Darya River.

### 3.2. Spatial and Vertical Variation in the Surface Soil Elements and Its Influencing Factors

**Figure 3:**
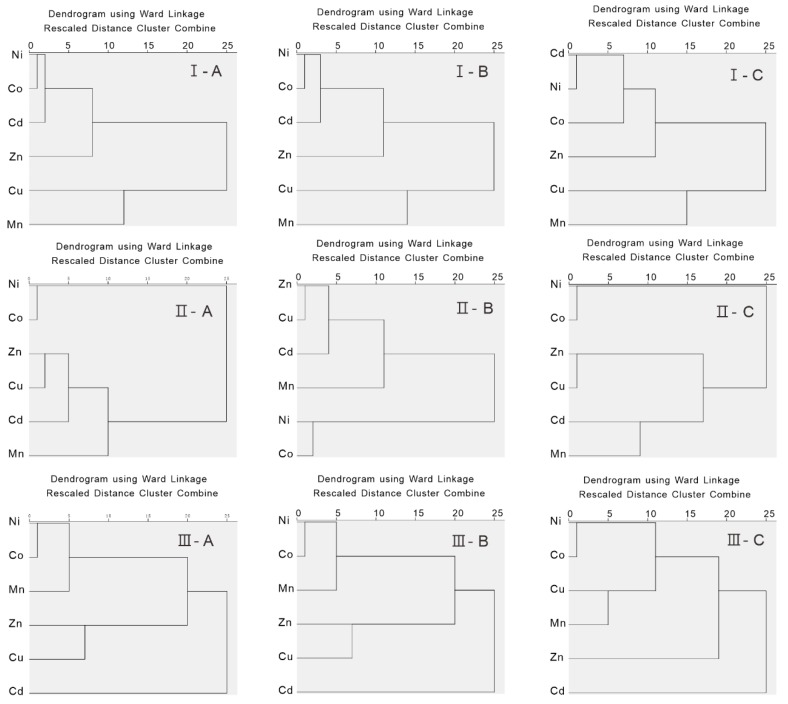
Of the cluster analysis data of the heavy metals in the three sampling regions (I: *n* = 29; II: *n* = 17; III: *n* = 29). A: The 0–20 cm soil layer; B: The 21–50 cm soil layer; and C: The 51–100 cm soil layer.

HCA was always used to analyze the interrelationships among the environmental variables [64,65,66]. The horizontal axis reflected the degree of the similarities among the heavy metals (Figure 3). Regarding the spatial variation, the similarity relationships among the elements in the three sampling regions differed significantly, which reflected significant differences in the soil formation environments (Figure 3). Cu and Mn in the surface layers of the first sampling region had similar characteristics, and Ni, Co, Cd, and Zn had similar variations. Co and Ni in the surface soils of the second region had similar variation characteristics, and Zn, Cu, Cd, and Mn had similar variation characteristics. In the surface soil of the third sampling region, Ni, Co, and Mn were similar, Cu and Zn were similar, and Cd was far from the other elements. Vertically, compared with the first layer A and the second layer B, the affinity distances of the three elements of Cd, Ni, and Co changed in the third layer C in the first sampling region I. For sampling region II, Co and Ni in the surface soils had similar variation characteristics in the three soil layers of A, B, and C, and Zn, Cu, Cd, and Mn had similar variation characteristics. The similarity characteristics of the heavy metals in the sampling region III changed obviously. Specifically, the surface layer and middle layer of the second and third sampling regions were basically the same, i.e., Ni, Co, and Mn had the close similarity, however, Ni, Co, Cu, and Mn were significantly clustered in the deep layer C. 

In the first sampling region I, the soil heavy metal elements were significantly correlated (Figure S1 in the supplementary file), reflecting that the heavy metals in the soil were mainly affected by the elemental composition of parent materials from which they are derived [9,67]. The coefficient of determination (R^2^) values of the linear regression equations between the contents of Cd, Co, Cu, Mn, Ni, and Zn in the surface soil and the corresponding heavy metals in the middle soil were 0.79, 0.91, 0.82, 0.92, 0.99, and 0.83, respectively. The coefficient of determination (R^2^) between the contents of Cd, Co, Cu, Mn, Ni, and Zn in the middle layer and the same heavy metal in the deep layers were 0.80, 0.82, 0.84, 0.89, 0.98, and 0.82, respectively. The coefficient of determination (R^2^) between the contents of Cd, Co, Cu, Mn, Ni, and Zn in the surface soils and the same heavy metal in the deep layers were 0.69, 0. 69, 0.73, 0.93, 0.98, and 0.63, respectively.

In sampling region II, the R^2^ values of the linear regression equations for the potentially toxic elements (Cu, Mn, and Zn) among the three different soil layers were greater than 0.5 (Figure S2 in the supplementary file). For two elements, Cd and Ni, although the R^2^ values of the surface and middle linear regression equations were 0.58 and 0.68, respectively, the R^2^ values of the linear regression equations between the middle and deep layers were only 0.29 and 0.12, respectively. There was no obvious linear relationship among the three layers for Co. This finding may indicate that the heavy metal elements in the upper and middle soils have been significantly affected by human activities.

In sampling region III, the soil heavy metal elements in the surface layers were significantly correlated with those in the middle layers; however, the R^2^ value of the linear regression equation for Cd between the contents in the middle layers and deep layer was 0.38, and the R^2^ the linear regression equation between the contents in the deep layers and top one was only 0.25 (Figure S3 in the supplementary file). Changes in the environmental factors in the process of soil formation in the late stage led to the composition of heavy metals in the upper and middle soil layers, which led to the deviation from the linear regression line.

### 3.3. Human Risk Assessment and Enrichment of Potentially Toxic Elements in the Surface Soils

From a global perspective, heavy metal pollution in soil caused by human activities has become increasingly serious [68,69]. According to existing studies in central Asia (e.g., the Lake Issyk-kul Basin [70] and the suburban region of Bishkek [71]), although the human health risk is low, it is affected by human activity. As shown in this paper, although the content of potentially toxic elements was not harmful to human health (Table 1, the thresholds of Zn, Cu, Cd, Ni, Co for HI = 1 were 107 × 10^3^, 14.4 × 10^3^, 254, 7.2 × 10^3^, and 6.2 × 10^3^ mg kg^−1^, respectively), it is also necessary to study the enrichment of potentially toxic elements caused by human activities.

**Figure 4:**
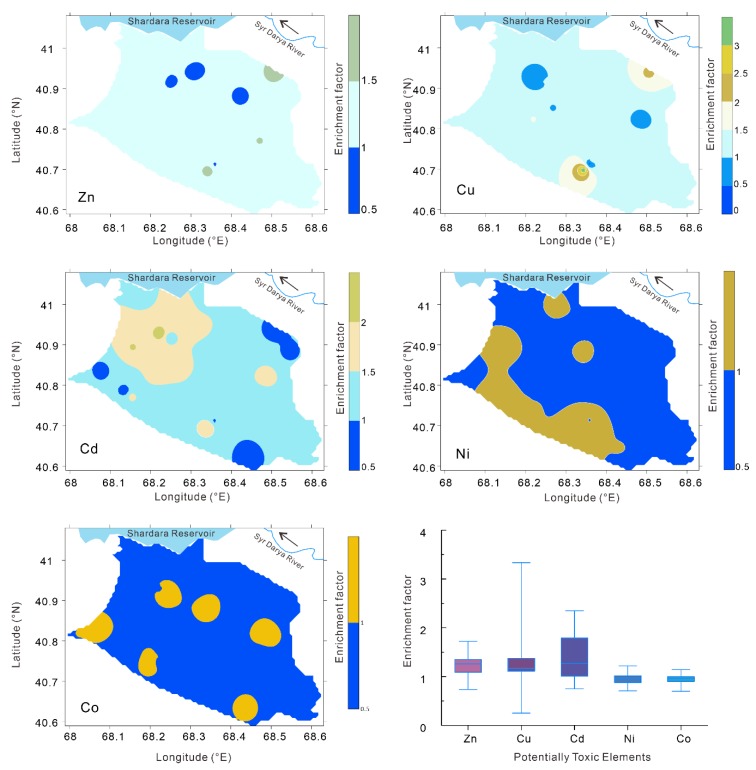
Basic statistical chart and spatial distribution maps of the enrichment coefficients of the potentially toxic elements in sampling region I.

In sampling region I, based on the statistical characteristics, the EFs of Zn, Cu, and Cd exceeding 1.5 accounted for 10.3%, 10.3%, and 34.5% of the total 29 soil profiles, respectively (Figure 4). Spatial variation in the EF of potentially toxic elements can be interpolated by inverse distance weighting [72]. According to the spatial distribution of the EFs of the potentially toxic elements, the EFs of Zn that were higher than one accounted for 97.62% of the total area, those exceeding 1.5 accounted for 1.16% of the total area, and there were no areas with EFs greater than two. The distribution areas of Cu and Cd with EFs greater than one accounted for 96.64% and 93.61% of the total area, the distribution areas greater than 1.5 accounted for 6.79% and 24.36% of the total area, and the distribution areas greater than two accounted for 1.15% and 0.51% of the total area, respectively. From the distribution maps of the EFs of Ni and Co, EFs between 1.0 and 1.5 accounted for 26.29% and 0.36% of the total area, respectively, and there were no areas where the EF exceeded 1.5.

**Figure 5:**
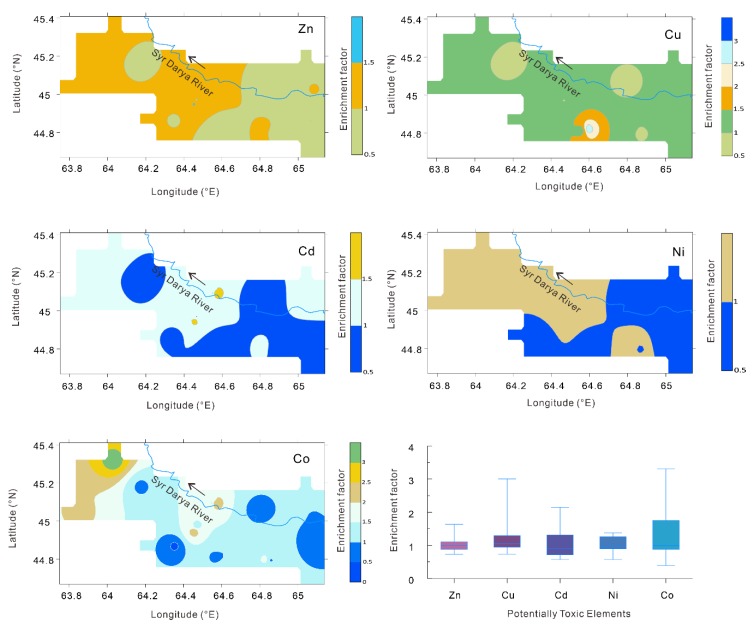
Basic statistical chart and spatial distribution maps of the enrichment coefficients of the potentially toxic elements in sampling region II.

In sampling region II, based on the statistical characteristics, the EFs of Zn, Cu, Cd, and Co exceeding 1.5 accounted for 5.9%, 5.9%, 11.8%, and 23.6% of the total 17 soil profiles, respectively (Figure 5). There was no EF of Ni in the soil exceeding 1.5. Moreover, the areas of Zn and Cd in the soil with EFs greater than 1.5 accounted for only 0.03% and 0.54% of the total area, respectively. For the potentially toxic elements Cu and Co, EFs greater than 1.5 accounted for 4.76% and 9.03% of the total area, respectively.

**Figure 6:**
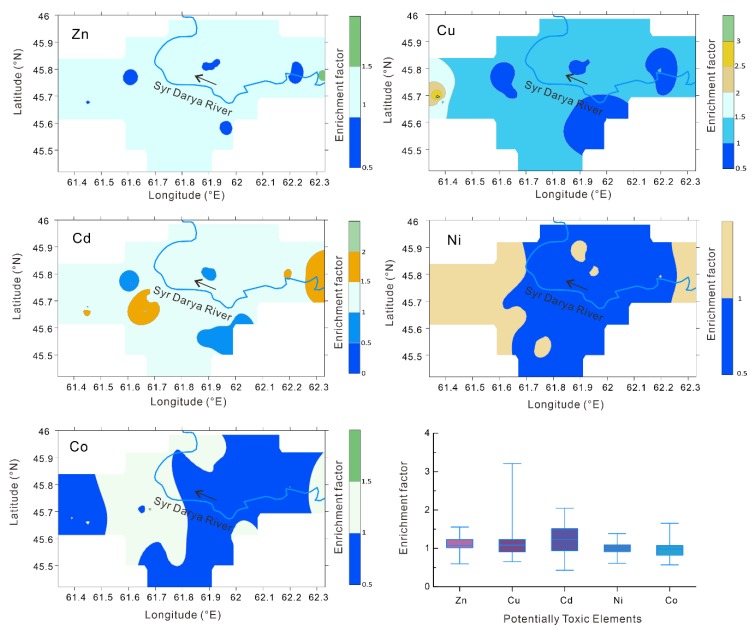
Basic statistical chart and spatial distribution maps of the enrichment coefficients of the potentially toxic elements in sampling region III.

In sampling region III, based on the statistical characteristics, the EFs of Zn, Cu, Cd, and Co exceeding 1.5 accounted for 10.3%, 6.9%, 27.6% and 3.4% of the total 29 soil profiles, respectively (Figure 6). There were no EFs of Ni and Co in the soil exceeding 1.5. The area of Zn in the soil with an EF greater than 1.5 accounted for only 0.24% of the total area. For the potentially toxic elements Cu and Cd, EFs greater than 1.5 accounted for 4.9% and 6.89% of the total area, respectively.

An EF value close to one indicates a natural source, a value less than one indicates the possible mobilization/depletion of a metal [49,73], and an EF value greater than one indicates that the element is affected by a human factor [49,74]. In theory, a heavy metal with an EF greater than one suggests that the heavy metal is mainly influenced by human activities [49,74,75,76]. However, there have been a number of studies suggesting that an EF lower than 1.5 reflects that a metal is mainly from crustal materials, whereas an EF value > 1.5 indicates non-natural weathering processes [77,78,79]. Combining the analysis in the previous section of this paper and the distribution of EFs in the region, it is more practical to use EF = 1.5 as a threshold for whether human activities affect heavy metal pollution in this region. Although the contents of the potentially toxic elements were not harmful to human health, the effects of human activities on the potentially toxic elements in irrigated soils revealed by EFs have been shown in this study area, which has resulted in a high degree of public concern.

## 4. Conclusions

By analyzing the heavy metals in the soil profiles in different irrigation areas of the Syr Darya River Basin, the spatial and vertical variation characteristics of heavy metals were determined, and the harm and enrichment of heavy metals to human health were evaluated. The conclusions are as follows.

(1) Environmental differences led to significant spatial differences in the similarities of the heavy metal elements in the soil. Based on element variation similarity revealed by HCA, the elemental groupings were consistent in the different layers only in region I. For regions II and III, the elemental groups in the surface soil layer and middle layer were basically the same but differed from those in the deep layer.

(2) In the first sampling region I, the heavy metals in surface soils were significantly correlated with the ones in deep layers, reflecting that they were mainly affected by the composition of parent materials. In the sampling region II, only Cu, Mn, and Zn showed significant correlations between the surface and deep layers. The similar phenomenon with significant correlation also existed heavy metals in the sampling region III, except for Cd.

(3) The contents of potentially toxic elements were not harmful to human health. It is more practical to use EF = 1.5 as the threshold for determining whether human activities affect heavy metal pollution in this region. The EFs of Zn, Cu, and Cd that were greater than 1.5 accounted for 1.16%, 6.79%, and 24.36% of sampling region I, respectively. In sampling region II, the EFs of Zn, Cu, Cd, and Co that were greater than 1.5 accounted for 0.03%, 4.76%, 0.54%, and 9.03% of the total area, respectively. In sampling region III, only the EFs of Zn, Cu and Cd that exceeded 1.5 accounted for 0.24%, 4.90%, and 6.89% of the total area, respectively.

## Figures and Tables

**Table 1:** Human health risk assessment of the potentially toxic elements in the surface soils of the Syr Darya River Watershed.

Sampling Region	Potentially Toxic Elements	Maximum Value (mg kg^−1^) ^a^	HQ_ing_^b^	HQ_inh_^c^	HQ_dermal_^d^	HI ^e^ = ΣHQ_i_
I	Zn	4.5	4.1 × 10^−5^	1.9 × 10^−9^	8.9 × 10^−7^	4.2 × 10^−5^
	Cu	2.8	1.9 × 10^−4^	9.0 × 10^−9^	2.8 × 10^−6^	2.0 × 10^−4^
	Cd	2.1	5.8 × 10^−3^	2.7 × 10^−7^	2.5 × 10^−3^	8.3 × 10^−3^
	Ni	28.7	3.9 × 10^−3^	1.9 × 10^−7^	6.3 × 10^−5^	4.0 × 10^−3^
	Co	13.1	1.8 × 10^−3^	3.0 × 10^−4^	9.8 × 10^−6^	2.1 × 10^−3^
II	Zn	4.8	4.4 × 10^−5^	2.1×10^−9^	9.5 × 10^−7^	4.5 × 10^−5^
	Cu	2.5	1.7 × 10^−4^	8.1 × 10^−9^	2.5 × 10^−6^	1.7 × 10^−4^
	Cd	1.6	4.4 × 10^−3^	2.1 × 10^−7^	1.9 × 10^−3^	6.3 × 10^−3^
	Ni	13.2	1.8 × 10^−3^	8.5 × 10^−8^	2.9 × 10^−5^	1.8 × 10^−3^
	Co	19.7	2.7 × 10^−3^	4.5 × 10^−4^	1.5 × 10^−5^	3.2 × 10^−3^
III	Zn	9.3	8.5 × 10^−5^	4.0 × 10^−9^	1.9 × 10^−6^	8.7 × 10^−5^
	Cu	4.9	3.4 × 10^−4^	1.6 × 10^−8^	4.9 × 10^−6^	3.4 × 10^−4^
	Cd	1.6	4.4 × 10^−3^	2.1 × 10^−7^	1.9 × 10^−3^	6.3 × 10^−3^
	Ni	17.5	2.4 × 10^−3^	1.1 × 10^−7^	3.9 × 10^−5^	2.4 × 10^−3^
	Co	22.3	3.1 × 10^−3^	5.1 × 10^−4^	1.7 × 10^−5^	3.6 × 10^−3^

^a^ The maximum values of potentially toxic elements in the surface layers were chosen as C_x_ in the formula (3), (4), and (5). ^b^ The hazard quotient (HQ) via ingestion. ^c^ The hazard quotient (HQ) via inhalation. ^d^ The hazard quotient (HQ) via dermal absorption. ^e^ Hazard index.

## Supplementary Materials

The following are available online at https://www.mdpi.com/1660-4601/16/22/4398/s1. Figure S1: Linear regressions for potentially toxic elements among three different layers in sampling region (I: *n* = 29), A: The 0–20 cm soil layer; B: The 21–50 cm soil layer; and C: The 51–100 cm soil layer. Figure S2: Linear regressions for potentially toxic elements among three different layers in sampling region (II: *n* = 17), A: The 0–20 cm soil layer; B: The 21–50 cm soil layer; and C: The 51–100 cm soil layer. Figure S3: Linear regressions for potentially toxic elements among three different layers in sampling region (III: *n* = 29), A: The 0–20 cm soil layer; B: The 21–50 cm soil layer; and C: The 51–100 cm soil layer. Table S1: The detailed information for sampling sites. Table S2: Descriptive statistical analysis of Mn and potentially toxic elements (Zn, Cu, Cd, Ni, Co, and Mn) in three sampling region (I: *n* = 29; II: *n* = 17; III: *n* = 29), A: The 0–20 cm soil layer; B: The 21–50 cm soil layer; and C: The 51–100 cm soil layer.
