# Supplementary material for: Spatial and Vertical Variations and Heavy Metal Enrichments in Irrigated Soils of the Syr Darya River Watershed, Aral Sea Basin, Kazakhstan
Source: Int J Environ Res Public Health. 2019 Nov 11;16(22):4398. doi: 10.3390/ijerph16224398 (PMC6888272; doi:10.3390/ijerph16224398)
Supplement: Supplementary file 1 [file ijerph-16-04398-s001.pdf]

Supplementary information

Table S1. The detailed information for sampling sites.

Series No.	Sampling Region	Sample sites	Longitude (°E)	Latitude (°N)	Altitude (m, a.s.l.)
1	I	I-01	68.50526	40.93862	250
2	I	I-02	68.5427	40.88215	254
3	I	I-03	68.46989	40.77136	256
4	I	I-04	68.42106	40.88129	251
5	I	I-05	68.22258	40.77501	258
6	I	I-06	68.21962	40.82377	252
7	I	I-07	68.15615	40.89438	255
8	I	I-08	68.34116	40.88617	260
9	I	I-09	68.30946	40.94336	251
10	I	I-10	68.28524	40.99123	249
11	I	I-11	68.33024	41.01403	251
12	I	I-12	68.18229	41.01555	253
13	I	I-13	68.22497	40.92811	254
14	I	I-14	68.24655	40.91841	256
15	I	I-15	68.26799	40.85138	256
16	I	I-16	68.02448	40.8186	253
17	I	I-17	68.07753	40.83648	250
18	I	I-18	68.13658	40.78587	251
19	I	I-19	68.15287	40.77223	258
20	I	I-20	68.20625	40.75551	257
21	I	I-21	68.2198	40.75059	260
22	I	I-22	68.36669	40.79713	259
23	I	I-23	68.48474	40.82255	257
24	I	I-24	68.5316	40.68704	256
25	I	I-25	68.43804	40.62501	262
26	I	I-26	68.433	40.63914	259
27	I	I-27	68.36541	40.71932	267
28	I	I-28	68.36026	40.71305	253
29	I	I-29	68.34389	40.69855	258
30	II	II -01	65.06782	44.87097	113
31	II	II -02	65.08489	44.87898	120
32	II	II -03	65.08376	45.02272	109
33	II	II -04	64.77037	45.06164	106
34	II	II -05	64.78937	45.06148	106
35	II	II -06	64.83265	44.79922	111
36	II	II -07	64.85312	44.79725	103
37	II	II -08	64.56181	44.81485	112
38	II	II -09	64.59298	44.82183	107
39	II	II -10	64.46661	44.96662	103
40	II	II -11	64.45846	44.94859	105
41	II	II -12	64.35402	44.86928	105
42	II	II -13	64.56777	45.09545	108
43	II	II -14	64.5568	45.09829	101
44	II	II -15	63.86495	45.04232	95
45	II	II -16	64.17681	45.18111	103
46	II	II -17	64.03373	45.31759	96
47	III	III-01	62.154	45.75127	61
48	III	III-02	62.32047	45.77691	62
49	III	III-03	62.21434	45.78647	59
50	III	III-04	62.19958	45.79304	62

51	III	III-05	61.9224	45.8772	49
52	III	III-06	61.90628	45.86677	52
53	III	III-07	61.91381	45.88637	57
54	III	III-08	61.92741	45.86905	55
55	III	III-09	61.6135	45.76378	58
56	III	III-10	61.93503	45.82127	54
57	III	III-11	61.94124	45.81637	58
58	III	III-12	61.88878	45.81199	55
59	III	III-13	61.91541	45.8027	52
60	III	III-14	61.97011	45.62403	64
61	III	III-15	61.99737	45.60885	55
62	III	III-16	61.97033	45.59065	50
63	III	III-17	61.88112	45.56472	54
64	III	III-18	61.74317	45.56526	49
65	III	III-19	61.72264	45.54898	58
66	III	III-20	61.75219	45.54571	60
67	III	III-21	61.67397	45.73242	62
68	III	III-22	61.67692	45.7079	55
69	III	III-23	61.65651	45.70687	52
70	III	III-24	61.38837	45.67584	46
71	III	III-25	61.66462	45.66462	49
72	III	III-26	61.44935	45.66517	49
73	III	III-27	61.37419	45.69549	58
74	III	III-28	61.45217	45.67813	56
75	III	III-29	61.45419	45.68829	57

Table S2. Descriptive statistical analysis of Mn and potentially toxic elements (Zn, Cu, Cd, Ni, Co and Mn) in three sampling region (I: n=29; II: n=17; III: n=29), A: the 0-20 cm soil layer; B: the 21-50 cm soil layer; and C: the 51-100 cm soil layer.

Region	Layer		Zn	Cu	Cd	Ni	Co	Mn
I	A	minimum	1.4	0.1	0.5	4.5	5.7	131.4
		maximum	4.5	2.8	2.1	28.7	13.1	195.3
		average	2.5	1.7	1.1	13.6	9.4	153.4
		median	2.4	1.7	1.0	7.5	9.1	147.7
		standard deviation	0.7	0.6	0.4	8.9	2.0	15.8
	B	minimum	1.4	0.2	0.5	4.5	6.2	134.9
		maximum	4.1	2.7	2.0	28.4	13.5	195.0
		average	2.5	1.7	1.1	14.1	9.6	154.2
		median	2.4	1.8	1.0	7.0	9.1	147.5
		standard deviation	0.6	0.5	0.4	8.6	2.1	15.7
	C	minimum	1.0	0.2	0.3	4.9	6.6	137.7
		maximum	3.6	2.2	2.6	29.4	16.7	193.0
		average	2.1	1.4	0.9	14.1	10.0	157.1
		median	2.0	1.5	0.8	7.8	9.6	150.2
		standard deviation	0.6	0.5	0.5	8.6	2.3	14.6
II	A	minimum	0.9	0.3	0.4	6.4	8.7	131.8
		maximum	4.8	2.5	1.6	13.2	19.7	333.7
		average	3.1	1.6	0.9	9.9	15.0	216.4
		median	3.3	1.9	0.9	9.7	16.3	211.5
		standard deviation	1.1	0.7	0.3	2.3	3.7	45.2
	B	minimum	0.6	0.1	0.2	3.7	3.6	90.2
		maximum	4.4	2.4	1.4	11.0	19.8	256.5
		average	2.5	1.2	0.8	8.2	10.6	174.3
		median	2.7	1.6	0.8	8.4	9.7	184.5
		standard deviation	1.0	0.7	0.3	2.1	4.0	47.1
	C	minimum	0.8	0.1	0.3	4.3	4.0	88.1
		maximum	4.8	1.9	1.5	12.0	20.8	291.8

		average	2.5	1.2	0.8	8.2	11.1	179.0
		median	2.4	1.3	0.7	7.8	9.0	185.0
		standard deviation	1.0	0.5	0.3	2.3	5.1	55.6
		minimum	0.7	0.5	0.2	0.7	2.9	63.8
		maximum	9.3	4.9	1.6	17.5	22.3	329.6
	A	average	4.0	2.4	0.8	8.8	13.3	199.2
		median	3.8	2.4	0.9	7.2	11.0	194.3
		standard deviation	2.1	0.9	0.3	4.9	6.1	62.7
		minimum	0.5	0.3	0.1	0.4	2.7	52.6
		maximum	8.0	3.4	1.2	17.1	22.0	321.7
III	B	average	3.4	2.0	0.6	7.7	11.8	173.0
		median	3.1	2.0	0.7	6.4	9.3	188.9
		standard deviation	1.9	0.7	0.3	4.5	5.9	61.5
		minimum	0.4	0.1	0.0	1.3	3.8	43.9
		maximum	8.5	3.5	1.4	18.0	22.1	326.9
	C	average	3.2	2.0	0.6	7.5	11.8	170.9
		median	2.7	1.9	0.6	6.0	8.9	169.5
		standard deviation	1.8	0.7	0.3	4.6	5.8	62.4
Guideline values of China [a]			300	100	250	60	-	-

[a] The value of guideline values of China (pH > 7.5, dry land soil) from the reference [1] Liu, G.; Yu, Y.; Hou, J.; Xue, W.; Liu, X.; Liu, Y.; Wang, W.; Alsaedi, A.; Hayat, T.; Liu, Z. An ecological risk assessment of heavy metal pollution of the agricultural ecosystem near a lead-acid battery factory. Ecol. Indic. 2014, 47, 210-218, doi:https://doi.org/10.1016/j.ecolind.2014.04.040.

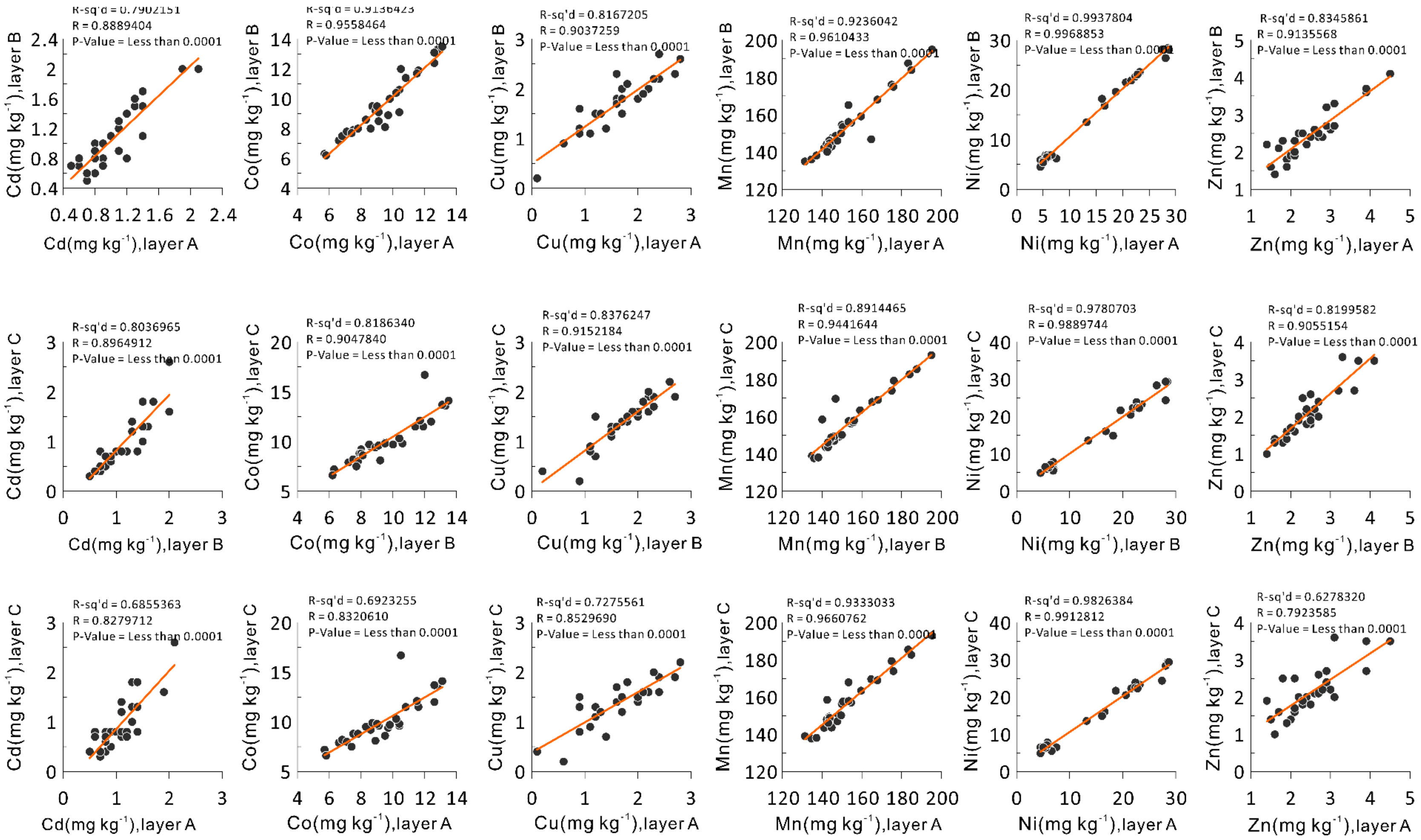

Figure S1. Linear regressions for Potentially Toxic Elements among three different layers in sampling region (I: n = 29), A: the 0-20 cm soil layer; B: the 21-50 cm soil layer; and C: the 51-100 cm soil layer.

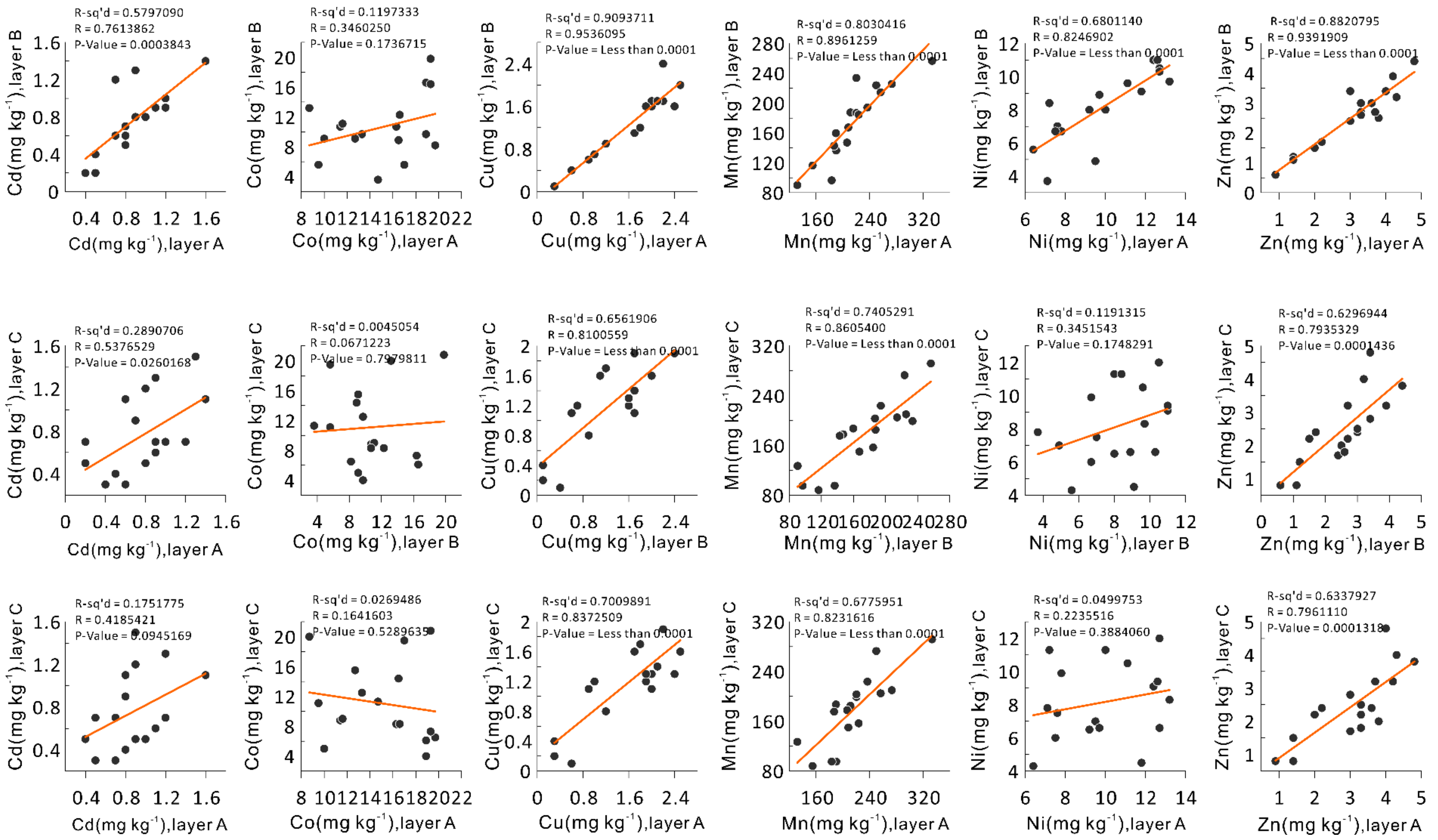

Figure S2. Linear regressions for Potentially Toxic Elements among three different layers in sampling region (II: n = 17), A: the 0-20 cm soil layer; B: the 21-50 cm soil layer; and C: the 51-100 cm soil layer.

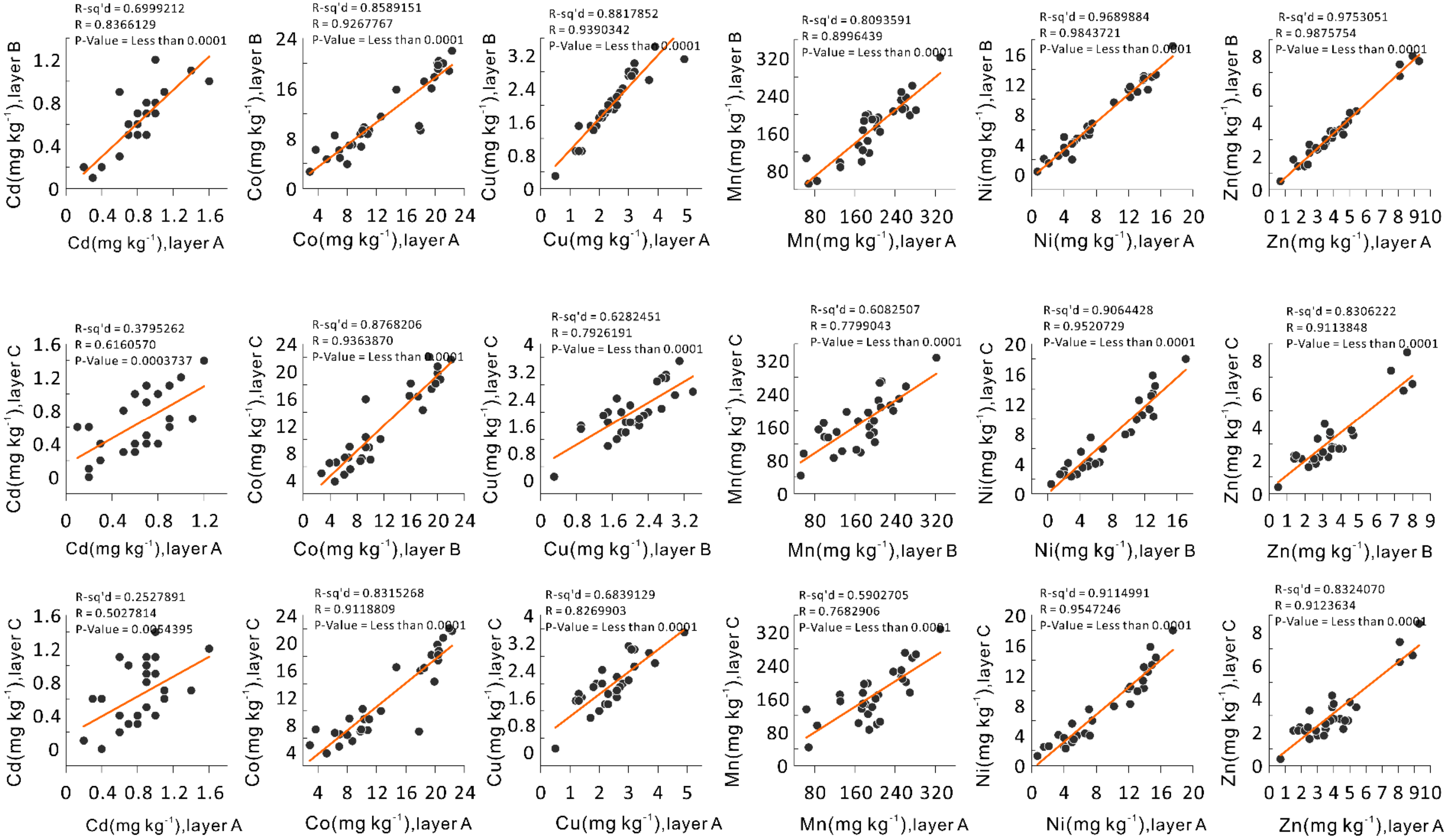

Figure S3. Linear regressions for Potentially Toxic Elements among three different layers in sampling region (III: n = 29), A: the 0-20 cm soil layer; B: the 21-50 cm soil layer; and C: the 51-100 cm soil layer.
